# Excising Parotid Benign Mass With a Minimal Incision

**DOI:** 10.7759/cureus.22382

**Published:** 2022-02-19

**Authors:** Fayez A A Alrohaimi, Muath Abuhaimed, Homood M Almutairi

**Affiliations:** 1 Otolaryngology - Head and Neck Surgery, Prince Sultan Military Medical City, Riyadh, SAU; 2 Otolaryngology - Head and Neck Surgery, King Saud Hospital, Qassim, SAU

**Keywords:** benign parotid tumor, head and neck cancer surgery, warthin's tumor, cosmetic incision, superficial parotidectomy

## Abstract

We report a case of a 30-year-old man who presented with a one-year history of a right-sided parotid mass that was asymptomatic but slowly increasing in size. On examination, there was a 2 × 2 cm superficial, soft, painless, and mobile mass in the right parotid region. Computed tomography revealed a benign mass in the right parotid tail measuring 2 × 3 cm, and a fine needle aspiration biopsy revealed a Warthin’s tumor. The patient was managed surgically using a new approach that involved complete excision of the mass via minimal cosmetic incision parotidectomy. Parotidectomy was performed using an incision that only involved the pre- and postauricular areas over the sulcus without any extensions, and the operation proceeded smoothly without any complications. The patient was discharged without postoperative complications

## Introduction

Parotid tumors are commonly encountered in the head and neck [1]. Approximately 3-6% of tumors encountered in these areas and 80% of parotid tumors are benign [2]. These tumors are usually asymptomatic and slow growing; however, if the tumor presents with symptoms such as otalgia, facial nerve paralysis, and rapid growth, malignancy is usually suspected [3,4]. Fine needle aspiration biopsy has high sensitivity and high specificity for identifying the characteristics of parotid tumors [5]. Imaging plays an important role in surgical planning because it assesses tumor location [6]. Parotidectomy is a common surgery because of the high incidence of parotid tumors. In any approach, good exposure is required to facilitate complete mass excision while preserving the facial nerve [1]. Parotidectomy is conventionally performed with a Blair incision or modified Blair incision (MBI), which involves a preauricular, postauricular, and extended incision to the lateral neck or hairline [7,8]. Because of the great potential impact of such post-parotidectomy facial scars on patients, a new method for performing the surgery was proposed. In this study, parotidectomy was performed using an incision that involved only the pre- and postauricular areas over the sulcus without any extensions. These incisions can produce the desired exposure in many cases and yield the desired surgical outcomes.

## Case presentation

The patient was a 30-year-old man with no medical history, who presented to the otolaryngology head and neck surgery clinic with a one-year history of a right-sided parotid mass that was slowly increasing in size. Tenderness, fluctuation, change in color, facial weakness, other masses, or constitutional symptoms were not noted. There was no previous history of radiation and no family history of the same condition.

On examination, there was a 2 × 2 cm superficial, painless, soft, and mobile mass in the right parotid region. The skin overlying the surface of the mass was normal with no discoloration or tethering. There was no cervical lymphadenopathy and no facial nerve paralysis. Direct flexible laryngoscopy findings were unremarkable.

**Figure 1:**
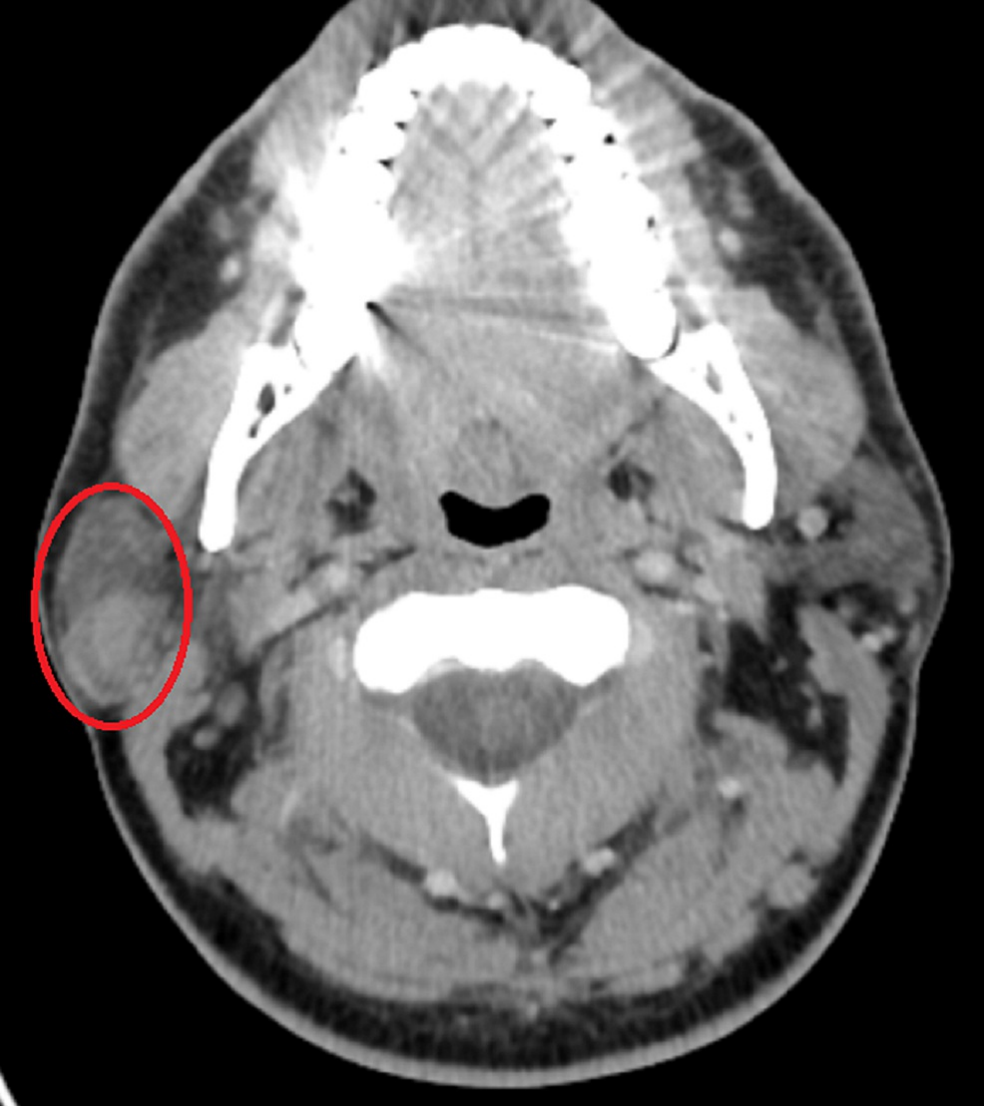
CT scan showing the tumor.

**Figure 2:**
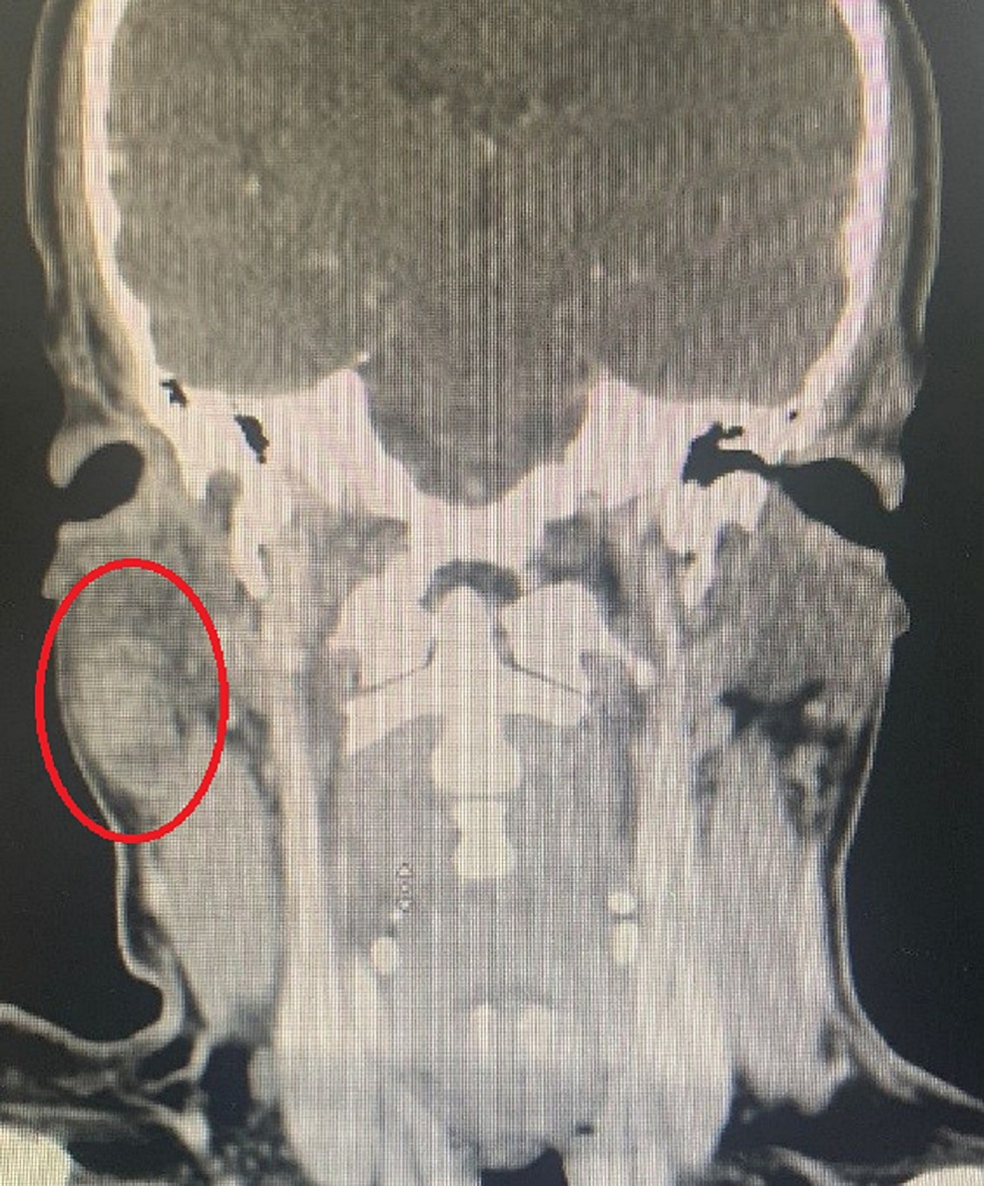
CT scan showing the tumor.

Contrast-enhanced computed tomography (CT) of the head and neck showed enhancement of a well-defined oval-shaped lesion in the right parotid gland in the superficial lobe with some exophytic component measuring 30 × 19 × 16 mm (Figures 1, 2).

Fine needle aspiration biopsy performed on smears showed cohesive, flat sheets of oncocytes. Oncocytes showed uniform, round nuclei with chromatin distribution, and the cytoplasm was rich and granular. Abundant reactive lymphocytes were observed scattered throughout the background. The features were consistent with a Warthin tumor.

Treatment

After discussing the benefits and possible complications of the surgery, as well as the possibility of avoiding a scar with the patient, the patient agreed to undergo surgery via our new approach. The patient was managed surgically with complete excision of the mass using minimal cosmetic incision parotidectomy. The operation proceeded smoothly without any complications, and there were no postoperative complications.

Surgical technique

The patient underwent surgery performed by a head and neck team under general anesthesia. The patient was lying supine with his neck extended while the head was turned around to the contralateral side. The incision was then initiated at the level of the right tragus in the pre-auricular region, proceeded along the skin crease, and proceeded downwards up to the ear lobe. It then turned around the ear lobe, proceeded posterosuperiorly along the post-auricular sulcus until the level of the right external auditory canal.

**Figure 3:**
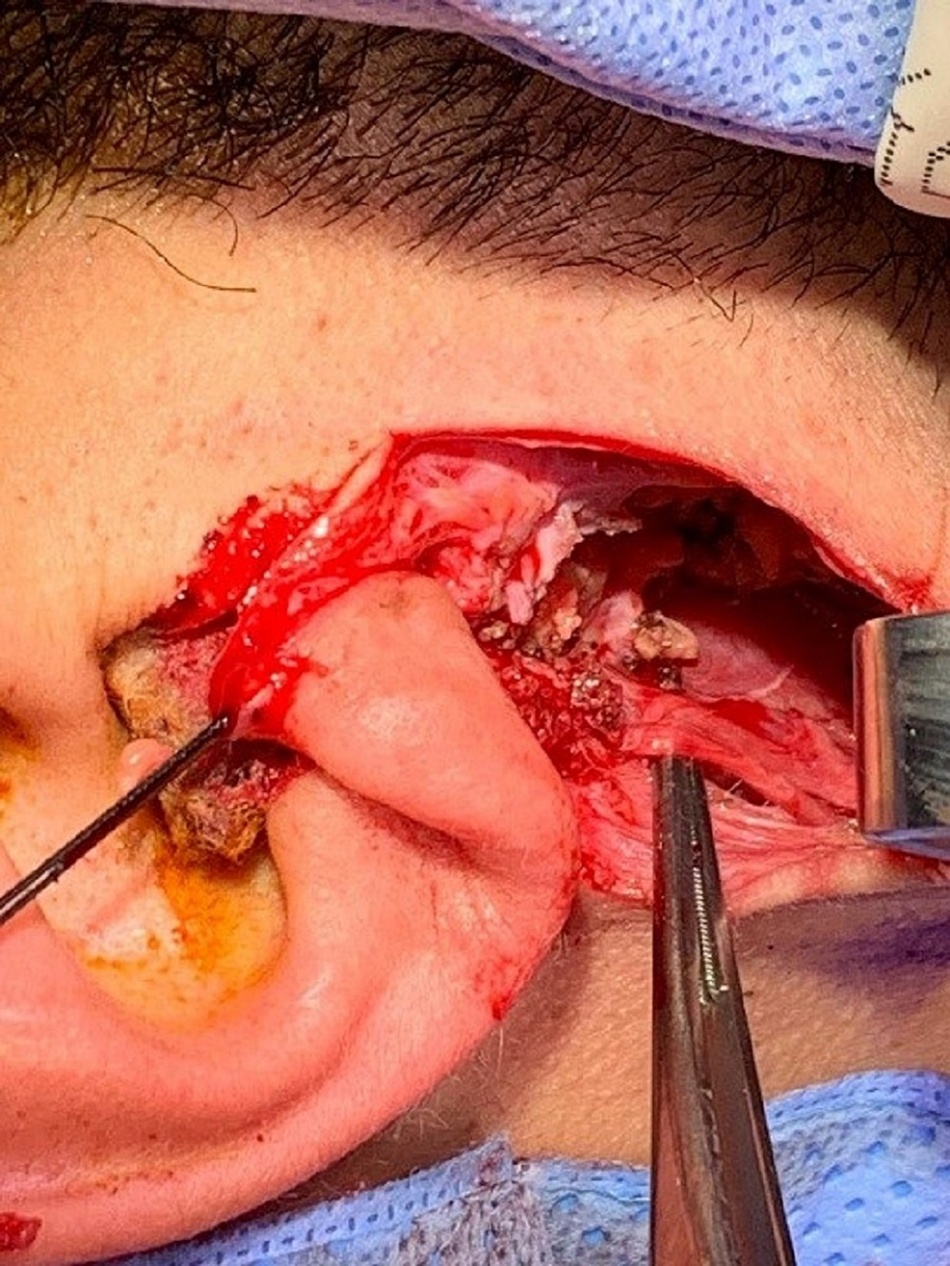
Intraoperative view of the incision.

**Figure 4:**
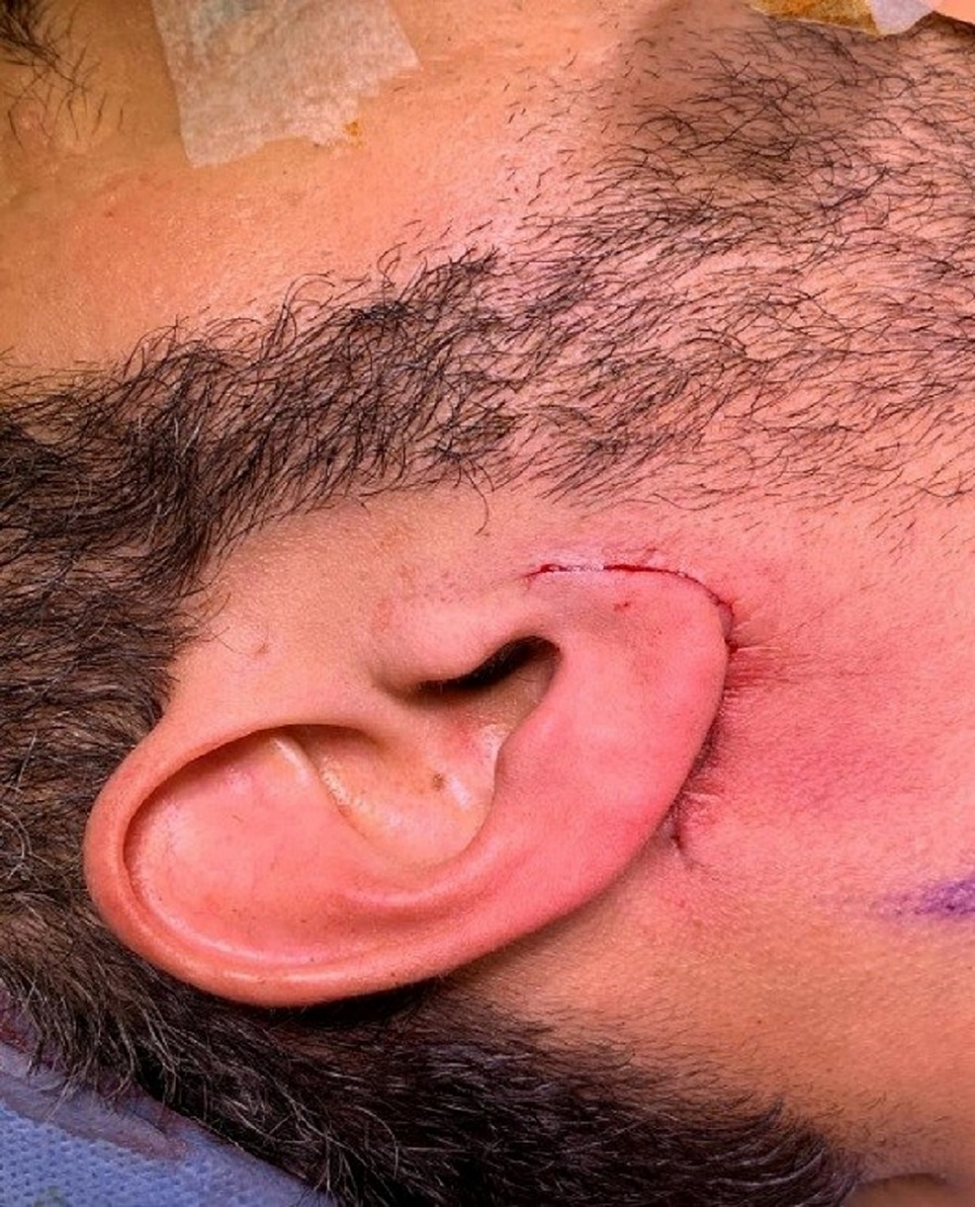
Patient’s scar post-surgery.

**Figure 5:**
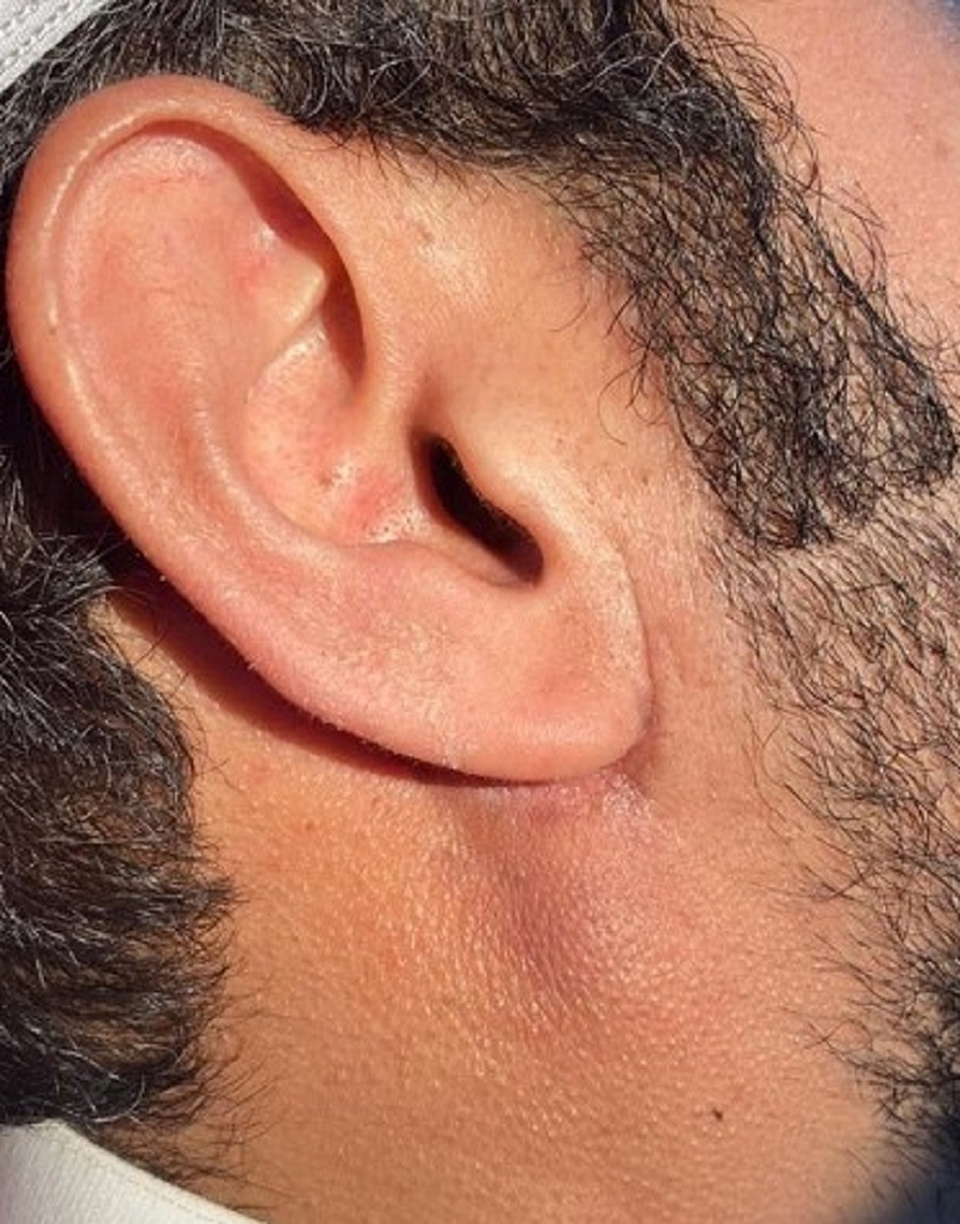
Patient’s scar two weeks post-surgery.

The skin flap was raised in three directions (anterior, inferior, and posterior) to show the right parotid gland and anterior border of the right sternocleidomastoid muscle. The ear lobe was separated from the parotid fascia then retracted superiorly. Dissection between the right parotid gland and right sternocleidomastoid muscle enabled identification of the facial nerve using the tympanomastoid fissure as a landmark. With adequate retraction of the flap, the relevant branch of the facial nerve was minutely dissected. The tumor was completely removed with a sufficient resection border that involved normal parotid tissue. A suction drain was then inserted in the posterior end of the incision. The wound was closed with interrupted 4-0 polyglactin 910 (Vicryl) sutures (Figures 3-5).

## Discussion

Parotid tumors are commonly encountered in the head and neck. Of parotid tumors, 80% are benign. Parotidectomy is a common surgery because of the high incidence of parotid tumors. Superficial parotidectomy is a common procedure used for both diagnosis and treatment. It is classically performed using an S-shaped Blair incision or facelift incision [1]. In the last two decades, the most common incision for parotidectomy has been the MBI. Despite the technique allowing for fast and wide exposure for the removal of parotid tumors, it has been linked to visible cervical scarring and retromandibular depression. Because of anesthetic outcomes, there has been the development of alternative approaches that are based on facelift incisions, such as the modified facelift incision, which has gained popularity due to its superiority with respect to cosmetics [7]. However, for better esthetic results, a V-shaped incision (VSI) is preferred in parotidectomy for benign parotid tumors. The VSI involves only the pre- and postauricular incisions, without any hairline or upper cervical inclusion. As a result, this method can be used to handle all superficial parotid regions, including both the superior and anterior divisions, while leading to minimal scarring [9].

The V-shape allows one to approach the entire superficial parotid region while minimizing the scar, as it eliminates any unnecessary parts for the incision. The VSI has been reported to have a lower mean operating time (120 min) and shorter drainage duration requirement (two to six days) than the facelift or Blair incisions, which have been found to require 150-180 min of operating time and three to four days of drainage [10]. In addition, the amount of drainage is lower than that with other methods, and this is evidence that the approach is less invasive and leads to minimal scarring. Unlike the modified facelift or retroarticular hairline incision, the VSI is not associated with necrosis of the skin flap or hypertrophic scarring [8]. Lastly, using these methods, patients are overall satisfied and happy with outcomes after surgery as compared to the other methods due to their non-invasive nature [1].

In a study by Ahn et al., 14 patients underwent VSI for variable benign parotid lesions. In all patients, the authors managed to remove the tumor while avoiding the paralysis of the facial nerve, flap necrosis, hematoma, hypertrophic scarring, and Frey’s syndrome [7].

Currently, one of the main limitations of this approach is the inability to excise tumors that arise from the parotid tail or extend inferiorly; however, this issue can be readily resolved with an extra hairline extension intraoperatively, which results in a modified facelift incision [11].

In our case, we are confident that the VSI achieved good exposure and a desirable cosmetic outcome with complete excision of the tumor.

## Conclusions

Parotid gland tumors account for 80% of all salivary gland neoplasms, and surgery aimed at removing these tumors is common. However, patients are increasingly cautious about their faces and want to undergo the most noninvasive method of operation. Given the noninvasive nature of the VSI approach, the purpose of these new methods is to achieve the best cosmetic and esthetic outcomes without affecting the complication rate of the procedure. In addition, our approach can be utilized because it achieves tumor resection while maintaining an acceptable esthetic outcome for the patient.
